# Uric acid is a risk factor for ischemic stroke and all-cause mortality in the general population: a gender specific analysis from The Tromsø Study

**DOI:** 10.1186/1471-2261-13-115

**Published:** 2013-12-11

**Authors:** Hilde M Storhaug, Jon V Norvik, Ingrid Toft, Bjorn O Eriksen, Maja-Lisa Løchen, Svetlana Zykova, Marit Solbu, Sarah White, Steve Chadban, Trond Jenssen

**Affiliations:** 1 Section of Haematology, University Hospital of North Norway, Tromsø, Norway; 2 Department of Clinical Medicine, UiT The Arctic University of Norway, Tromsø, Norway; 3 Section of Nephrology, University Hospital of North Norway, N-9038, Tromsø, Norway; 4 Department of Community Medicine, UiT The Arctic University of Norway, Tromsø, Norway; 5 Renal Division, The George Institute for International Health, University of Sydney, Sydney, Australia; 6 Renal Medicine, Royal Prince Alfred Hospital, Camperdown, Sydney, Australia; 7 Department of Nephrology, Oslo University Hospital Rikshospitalet, Oslo, Norway

**Keywords:** All-cause mortality, Gender, Myocardial infarction, Stroke, Uric acid

## Abstract

**Background:**

The role of serum uric acid as an independent predictor of cardiovascular disease and death is uncertain in the general population. Adjustments for additional cardiovascular risk factors have not been consistent. We examined the association of serum uric acid with all-cause mortality, ischemic stroke and myocardial infarction in a prospective population based study, with several traditional and non-traditional risk factors for cardiovascular disease included in the model.

**Methods:**

A population-based prospective cohort study was performed among 2696 men and 3004 women. Endpoints were all-cause mortality after 15 years, and fatal or non-fatal myocardial infarction (MI) and ischemic stroke after 12 years.

**Results:**

1433 deaths, 659 MIs and 430 ischemic strokes occurred during follow-up. Fully adjusted Cox regression analyses showed that per 1 SD (87 μmol/L) increase in serum uric acid level, the risk of all-cause mortality increased in both genders (hazard ratios, HR men; 1.11, 95% CI 1.02-1.20, women; 1.16, 1.05-1.29). HRs and 95% CI for stroke were 1.31, 1.14-1.50 in men, 1.13, 0.94-1.36 in women, and 1.22 (1.09, 1.35) in the overall population. No independent associations were observed with MI.

**Conclusion:**

Serum uric acid was associated with all-cause mortality in men and women, even after adjustment for blood pressure, estimated GFR, urinary albumin/creatinine ratio, drug intake and traditional cardiovascular risk factors. After the same adjustments, serum uric acid was associated with 31% increased risk of stroke in men.

## Background

Uric acid is the breakdown product of purines from DNA, RNA, ATP and cAMP. In this process hypoxanthine is converted by the enzyme xanthine oxidase to xanthine and further to uric acid. Both steps induce the release of free radicals. Uric acid may accumulate in the body due to increased production (cell death, intake of alcohol or purine rich diet) or decreased elimination (impaired renal function, use of diuretics). Epidemiologic studies show that mean uric acid levels in men increased gradually from the 1920s to the 1970s, from less than 210 μmol/L to 360-390 μmol [1]. Pre-menopausal women tend to have lower levels than men, probably because of the uricosuric effect of estrogens [2]. The relationship between serum uric acid and cardiovascular disease is not clear. Some epidemiologic studies have reported a relationship between serum uric acid and several cardiovascular conditions [3-10], whereas others have not observed such links [11-15]. The National Health and Nutrition Examination Survey (NHANES I study) [3] reported a significant risk for cardiovascular death with increasing serum uric acid levels. The Framingham Heart study, on the other hand, was not able to confirm these findings when use of diuretics was adjusted for [11]. Two large cohort studies from Japan and Korea, each with 9-10 years follow up could not confirm that serum uric acid was a risk factor for either cardiovascular disease or death [12,13]. It has been argued that previous studies may not have sufficiently accounted for differences in gender or for risk factors being strongly related to serum uric acid levels, e.g., use of diuretics or renal factors, such as glomerular filtration rate (GFR) and renal dysfunction measured as urinary albumin excretion.

The purpose of the present study was to address uric acid as putative cardiovascular risk factor in a Caucasian population followed for more than 12 years. In order to do so, we chose a stepwise approach with models including various covariates that have and have not been included in previous studies.

## Methods

### Study population

**Figure 1:**
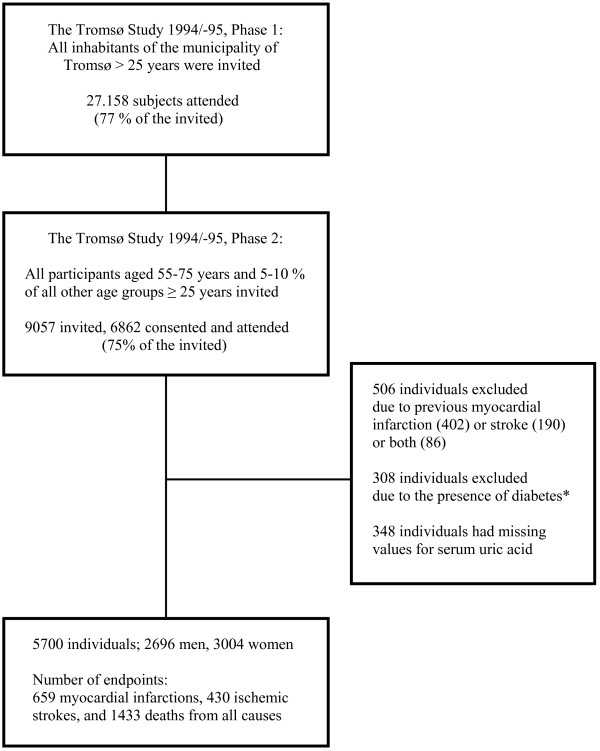
**Selection of the study population.** * Self-reported diabetes mellitus and/or use of antidiabetic medication and/or HbA1c > 6.5% and/or non-fasting glucose ≥ 10.0 mmol/L.

The Tromsø Study is a series of population-based, prospective surveys of inhabitants of the municipality of Tromsø, Norway [16]. In 1994/95, 27.158 subjects were screened (77% of eligible subjects). All participants at the age of 55-75 years and 5-10% of the other age groups ≥ 25 years were invited to a second visit including a more comprehensive examination 4-6 weeks later. Of the 9057 individuals who were invited 6862 participated (attendance rate 75%). Persons with known previous MI (n = 402), ischemic stroke (n = 190) or diabetes (n = 308), defined as self-reported diabetes, use of antidiabetic medication, HbA1c > 6.5% or non-fasting plasma glucose ≥10.0 mmol/L, were excluded. Data on uric acid was available in 5700 subjects (Figure 1). The Tromsø Study was conducted by the University of Tromsø in cooperation with The National Health Screening Service. The Regional Committee for Medical Research Ethics approved the study, and all participants gave their written consent.

### Measurements

All measurements and information on risk factors were obtained from baseline data of the 4^th^ Tromsø Study in 1994 /95. Information about presence of diabetes, smoking habits and physical activity was obtained from a self-administered questionnaire. Blood pressure was recorded in triplet (Dinamap) after 5-min seating, the mean of the second and third measurement was used. Hypertension was defined as systolic blood pressure (SBP) ≥ 140 mmHg and/or diastolic pressure (DBP) ≥ 90 mmHg and/or current use of antihypertensive medication. Physical activity was classified as active (> 1 hour physical activity/week) or inactive (all others). Smoking habits were classified as non-smokers or current smokers. Serum HDL-cholesterol was measured after precipitation of lower-density lipoprotein with heparin and manganese chloride. Serum uric acid was measured by photometry with COBAS® instruments (Roche diagnostics, Switzerland) using an enzymatic colorimetric test, the uricase/ PAP method. Reference values were 140-340 μmol/L (2.4-5.7 mg/100 mL) for females and 200-415 μmol/L (3.4-7.0 mg/100 mL) for males. Creatinine was analyzed by a modified Jaffe reaction, but since creatinine-based estimation of GFR is better validated for enzymatic creatinine measurements, 111 plasma samples from the 1994/95 survey were thawed and reanalysed with an enzymatic method (Modular P/Roche). Values were fitted to a linear regression model, and recalibrated creatinine values were calculated for all participants. Estimated GFR was calculated according to the CKD-EPI formula [17]: eGFR = 141 × min(S_Cr_/k,1)^a^ × max(S_Cr_/k,1)^-1.209^ × 0.993^age^ × ([1.018 if female] and × [1.159 if black]) where S_Cr_ is serum creatinine (mg/dL), k is 0.7 for females and -0.411 for males, min indicates the minimum of S_Cr_/k and max indicates the maxiumum of S_Cr_/k). Albuminuria was reported as albumin/creatinine ratio (ACR, mg/mmol, urinary albumin and creatinine analyses; kits from ABX Diagnostics, Montpellier, France). For each subject, ACR was measured in fresh samples of each of 3 separate urine specimens and the mean of all 3 was used in the analyses.

### Outcomes

The endpoints were death from any cause, first ever non-fatal or fatal myocardial infarction or ischemic stroke. Adjudication of hospitalized and out-of-hospital events was performed by an independent endpoint committee, who thoroughly reviewed data from hospital and out-of-hospital journals, autopsy records and death certificates. Event ascertainment followed a detailed protocol according to established diagnostic criteria. Each case was reviewed separately. Stroke was defined according to the WHO definition, only ischemic strokes were included [18]. Individuals who had died, moved, or emigrated from Tromsø were identified through the population Registry of Statistics Norway. The national 11-digit identification number allowed a linkage to the population Registry of Statistics Norway and ensured a complete follow-up status for all-cause mortality until November 30^th^, 2010 (follow-up time 15 years). The cardiovascular (CV) endpoint registry was completed until December 31, 2007 (follow-up time 12 years). Data were censored for date of registered emigration, or deaths from causes other than myocardial infarction and stroke.

### Statistics

Data are given as mean ± SD or median and interquartile range. Uric acid was categorized into gender-specific tertiles. Crude and age-adjusted incidence rates were calculated as events per 1000 person years at risk. Age-adjustment of incidence rates was performed on 10 year age groups with the population of Tromsø in 1995 as the standard population. Multiple linear regression analyses were performed with uric acid as the dependent variable. Covariates were systolic blood pressure, body mass index (BMI), high density lipoprotein cholesterol (HDL), total cholesterol, smoking status and physical activity. Renal covariates (estimated GFR and ACR) and use of diuretics or antihypertensive drugs were also added to the models in the multivariable analyses. ACR was logarithmically transformed. Cox proportional hazard models were used to investigate associations of uric acid with cardiovascular outcomes and mortality, calculated per 1 SD (87 μmol/L) change in uric acid, in unadjusted, age-adjusted and multivariable analyses. The proportional hazard assumption was checked by visual inspection of the -log-log survival curves. Tests for interactions and non-linearity (by quadratic terms) were assessed in separate models. Non-linear effects were also explored in fractional polynomial regression models. P values < 0.05 were considered statistically significant. Most analyses were run using SPSS software version 15.0 (SPSS, INC, Chicago, Illinois), fractional polynomial regression models were performed with STATA/MP 12.1 (Stata Corp LP, College Station, Texas).

## Results

### Baseline characteristics

**Figure 2:**
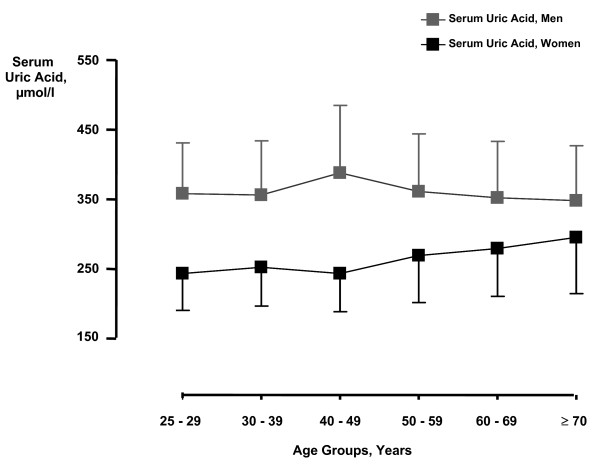
**Serum uric acid levels by gender and age groups.** Mean (SD).

Mean serum uric acid was 357 ± 84 μmol/L for men and 276 ± 70 μmol/L for women. Figure 2 shows serum uric acid concentrations according to gender and age. In both genders, increasing uric acid was associated with a poorer risk profile in terms of elevated BMI, blood pressure, ACR, and proportion of hypertensive persons. Physical activity, renal function and HDL were lower with increasing uric acid concentrations. Only a few subjects were using diuretics and anti-hypertensive medication at baseline in 1994/95 (Table 1).

**Table 1:** Baseline characteristics according to gender and serum uric acid tertiles

**Men**				
SUA tertiles	I ≤ 317μmol/L	II 318-380 μmol/L	III 381-976 μmol/L	P value for linear trend
N	896	902	898	
Age, years	60 ± 10	59 ± 10	59 ± 11	<0.001
Serum uric acid (SUA), μmol/L	276 ±33	348 ± 18	449 ± 70	-
BMI, kg/m^2^	24.7 ± 2.9	25.8 ± 3.0	27.3 ± 3.4	<0.001
SBP, mm Hg	144 ± 20	144 ± 20	146 ± 20	<0.001
DBP, mm Hg	84 ± 12	85 ± 12	86 ± 12	<0.001
Hypertension, n (%)	482 (54%)	546 (61%)	571 (64%)	<0.001
Total cholesterol, mmol/l	6.35 ± 1.19	6.40 ± 1.20	6.73 ± 1.20	<0.001
HDL cholesterol, mmol/l	1.51 ± 0.40	1.40 ± 0.39	1.26 ± 0.38	<0.001
GFR ckd-epi, ml/min/1.73m^2^	96.7 (90.9, 102.4)	95.5 (88.0, 102.5)	93.4 (84.0, 101.5)	<0.001
ACR mg/mmol	0.55 (0.38, 0.96)	0.51 (0.33, 1.01)	0.54 (0.34, 1.13)	0.021
Use of antihypertensive drugs, n (%)	74 (8%)	87 (10%)	155 (17%)	<0.001
Use of diuretics , n (%)	2 (0.2%)	5 (0.6%)	13 (1.4%)	<0.001
Current smoker, n (%)	351 (39%)	300 (33%)	294 (33%)	<0.001
Physically active, n (%)	301 (33%)	268 (30%)	244 (27%)	<0.001
**Women**				
SUA tertiles	I ≤ 244 μmol/L	II 245-297 μmol/L	III 298-681 μmol/L	P value for linear trend
N	1002	1001	1001	
Age, years	58 ± 11	60 ± 10	62 ± 9	<0.001
Serum uric acid (SUA), μmol/L	206 ± 28	269 ± 15	354 ± 54	-
BMI, kg/m^2^	24.0 ± 3.5	25.6 ± 3.8	27.7 ± 4,6	<0.001
SBP, mm Hg	138 ± 23	143 ± 23	151 ± 25	<0.001
DBP, mm Hg	78 ± 13	81 ± 12	85 ± 14	<0.001
Hypertension, n (%)	446 (45%)	534 (53%)	679 (68%)	<0.001
Total cholesterol, mmol/l	6.63 ± 1.39	6.82 ± 1.29	7.14 ± 1.32	<0.001
HDL cholesterol, mmol/l	1.76 ± 0.43	1.69 ± 0.43	1.54 ± 0.42	<0.001
GFR ckd-epi, ml/min/1.73m^2^	96.9 (90.5,103.0)	93.6 (86.0, 99.4)	89.7 (79.4, 97.1)	<0.001
ACR, mg/mmol	0.62 (0.41, 0.96)	0.58 (0.41, 0.93)	0.64 (0.42, 1.14)	<0.001
Use of diuretics, n (%)	3 (0.3%)	7 (0.7%)	39 (3.9%)	<0.001
Use of antihypertensive drugs, n (%)	65 (6.5%)	107 (11%)	148 (15%)	<0.001
Current smoker, n (%)	339 (34%)	315 (32%)	278 (28%)	0.001
Physically active, n (%)	162 (16%)	177 (18%)	118 (12%)	<0.002

The Tromsø Study 1994/95.

BMI: Body mass index. SBP: Systolic blood pressure. DBP: Diastolic blood pressure Hypertension: SBP ≥140 mmHg and/or DBP ≥90 mmHg and/or self-reported use of antihypertensive medication. Physical active: Self-reported hard physical activity ≥1 hr per week (yes / no).

GFR: Glomerular Filtration Rate according to the CKD EPI formula [17]. ACR: Urinary albumin/creatinine ratio (mean of 3 urine samples).

### The association of serum uric acid with renal function and CV risk factors

The correlation between uric acid and age differed in men and women (men; r = -0.09, p < 0.001, women; r = 0.17, p < 0.001, Pearson’s correlation coefficients), and tested significant for gender interaction (P < 0.001). Results of the multiple linear regression analyses are given in Table 2. In both genders, standardized beta-coefficients were highest for BMI, HDL-cholesterol, total cholesterol, and GFR. The addition of renal factors (eGFR and ACR; model 5) into the model including drug intake and traditional cardiovascular risk factors, contributed significantly to variation of serum uric acid; adjusted R^2^ increased from 0.19 to 0.23 in men, from 0.20 to 0.27 in women, and from 0.20 to 0.40 in pooled analyses for men and women together. ACR was independently associated with serum uric acid in women, but not in men, and there was a significant interaction with gender (P = 0.005).

**Table 2:** Multiple regression analysis with cardiovascular and renal covariants, and serum uric acid as dependent variable

		**Men n = 2696**		**Women n = 3004**		**Men and women n = 5700**			
	β (95% CI*)	**Std. β-coeffcent.**^**ϯ**^	**P-value**	β (95% CI*)	**Std. β-coeffcent.**^**ϯ**^	**P-value**	β (95% CI*)	**Std. β-coeffcent.**^**ϯ**^	**P-value**
**Model 1**									
Intercept	175 (143, 208)	-	<0.001	71 (52, 90)	-	<0.001	128 (108, 148)	-	<0.001
Age	-0.70 (-0.10, -0.39)	-0.09	<0.001	0.55 (0.30, 0.80)	0.08	<0.001	-0.39 (-0.61, -0.17)	-0.05	0.001
BMI	8.29 (7.38, 9.21)	0.33	<0.001	5.31 (4.73, 5.88)	0.32	<0.001	6.79 (6.21, 7.37)	0.30	<0.001
SBP	0.06 (-0.10, 0.21)	0.01	0.49	0.24 (0.13, 0.35)	0.08	< 0.001	0.24 (0.13, 0.35)	0.06	<0.001
Adjusted R^2^	0.12			0.15			0.10		
**Model 2**									
HDL-cholesterol	-44.42 (-52.09, -36.76)	-0.22	<0.001	-27.68 (-33.12,-22.23)	-0.17	<0.001	-61.58 (-66.42, -56.74)	-0.32	<0.001
Cholesterol	10.12 (7.70, 12.54)	0.15	<0.001	6.38 (4.50, 8.26)	0.12	<0.001	6.33 (4.67, 7.99)	0.10	<0.001
Adjusted R^2^		0.17		0.18			0.19		
**Model 3**									
Antihypertensive									
Drugs	26.10 (16.76, 35.44)	0.10	<0.001	14.19 (6.55, 21.83)	0.06	0.001	20.37 (13.66, 27.08)	0.07	<0.001
Diuretics	66.90 (33.13, 99.98)	0.07	<0.001	69.14 (51.01, 87.28)	0.13	<0.001	54.52 (35.75, 73.29)	0.07	<0.001
Adjusted R^2^	0.18			0.20			0.20		
**Model 4**									
Current smoker	-7.73 (-13.81, -1.63)	-0.04	0.01	2.07 (-3.02, 7.16)	0.01	0.4	-0.80 (-5.23, 3.62)	-0.004	0.7
Physical activity	-7.29 (-13.56, -1.02)	-0.04	0.02	1.17 (-5.27, 7.61)	0.01	0.7	11.28 (6.35, 16.20)	0.05	<0.001
Adjusted R^2^	0.19			0.20			0.20		
**Model 5**									
GFR ckd-epi	-1.76 (-2.05, -1.48)	-0.28	<0.001	-1.73 (-1.94, -1.51)	-0.32	<0.001	-1.74 (-1.91,-1.56)	-0.27	<0.001
logACR	4.09 (-2.58, 10.76)	0.02	0.23	15.41 (9.28, 21.55)	0.08	< 0.001	8.09 (3.58, 12.61)	0.04	<0.001
Gender	- -	-	-	- -	-	-	76.87 (73.08, 80.66)	0.44	<0.001
Adjusted R2	0.23			0.27			0.40		

*Confidence interval. ^ϯ^Standarized β-coeffcient. Gender: 1 = male, 0 = female, GFR: Glomerular filtration rate according to the CKD EPI formula [17].

ACR: urinary albumin/creatinine ratio (mean of 3 urine samples) logACR: logarithmic transformation of ACR. Model 1: Age, body mass index.

(BMI), systolic blood pressure (SBP). Model 2: Model 1 + HDL-cholesterol and total cholesterol. Model 3: Model 2 + use of diuretics and other anti-hypertensive medication. Model 4: Model 3 + current smoking and physical activity (self-reported hard physical activity ≥1 hr per week; yes = 1 / no = 0), current smoking (yes = 1 / no = 0). Model 5: Model 4 + renal factors added (GFR the CKD-EPI and logACR).

### Event rates

**Figure 3:**
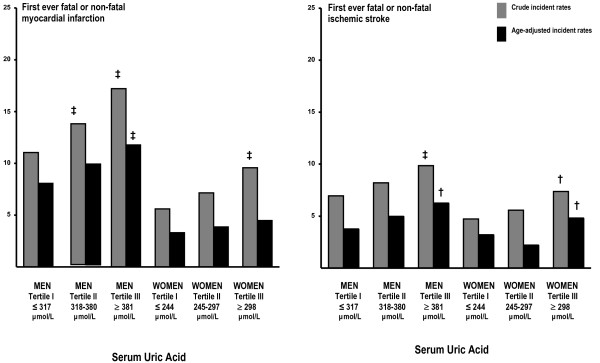
**Crude and age-adjusted incidence rates by serum uric acid tertiles.** † : P < 0.05. ‡ : P < 0.001 (differences between tertiles were tested for using a normal test with continuity correction, tertile 1 was reference).

Number of events during follow-up were 659 first ever cases of fatal or non-fatal MI, 430 fatal or non-fatal ischemic strokes, and 1433 deaths from all causes. Median observation time was 12.5 years for myocardial infarction and ischemic stroke, and 15.7 years for all-cause mortality. Crude and age-adjusted incidence, for MI and ischemic stroke, picturing absolute risk rates according to increasing tertiles of serum uric acid, are shown in Figure 3. Age-adjusted incidence rate for myocardial infarction was significantly higher in the upper serum uric acid tertile among men, but not in women. Increasing crude and age-adjusted incidence rates with higher levels of uric acid were observed in both genders for ischemic stroke.

### Associations between serum uric acid and outcome variables (Cox proportional hazard models)

In both genders, P-values for linear, increasing trend were <0.001 for all endpoints in multivariable, Cox proportional hazard models where the lowest serum uric acid tertile was reference (data not shown). In multivariable analyses calculated per 1 SD (87 μmol/L) increase in uric acid (Table 3), uric acid was independently associated with all-cause mortality in men (HR, 95% CI; 1.11, 95% CI 1.02-1.20), women (HR, 95% CI; 1.16, 1.05-1.29), and in the total population (men and women; HR, 95% CI: 1.13, 1.06-1.21). In fully adjusted, multivariable models, serum uric acid was not independently associated with MI in any gender or in the mixed population. In both genders, uric acid lost significance when lipids were introduced as covariates. Serum uric acid was strongly associated with ischemic stroke in men after multivariable adjustments (HR, 95% CI: 1.31, 1.14-1.50). In contrast, significance was lost among women after adjustments for blood pressure and BMI. Test for gender interaction was not significant (P = 0.19). In multivariable analysis of the mixed population, adjusted for sex, 87 μmol/L increase in uric acid was associated with a 24% increase in risk of future ischemic stroke (Table 3). Tests for non-linearity in fractional polynomial models for associations of serum uric acid with the three endpoints were not significant in any gender. Cox regression analyses were also performed without exclusion of persons with diabetes and previous cardiovascular disease. Risk estimates for uric acid and myocardial infarction in fully adjusted analyses did not change substantially (HR and 95% CI for men were 1.06, 0.99-1.14, p = 0.06 and for women 1.04, 0.92-1.14, p = 0.5). We also stratified the study population according to serum uric acid levels higher or lower than the hospital laboratory’s upper reference range. Belonging to the group with higher uric acid levels was not significantly associated with myocardial infarction in men (P = 0.26) or women (P = 0.27) after multivariable adjustments.

**Table 3:** Associations of serum uric acid with adverse cardiovascular events and all-cause mortality

	**Hazard ratio (95% CI) per 1 SD increase in serum uric acid (87 μmol/L)**					
	**Men (n = 2696)**	**P-value**	**Women (n = 3004)**	**P-value**	**Men and women ( n = 5700)**	**P-value**
**All-cause mortality**						
Unadjusted	1.01 (0.94, 1.09)	0.6	1.24 (1.14, 1.35)	<0.001	1.10 (1.04, 1.17)	<0.001
Age-adjusted	1.11 (1.03, 1.19)	0.005	1.12 (1.03, 1.22)	0.008	1.11 (1,05, 1.18)	<0.001
Multivariable adjustment						
Model 1	1.13 (1.04, 1.22)	0.003	1.22 (1.12, 1.33)	<0.001	1.19 (1.14, 1.25)	<0.001
Model 2	1.13 (1.05, 1.23)	0.003	1.20 (1.10, 1.31)	0.001	1.17 (1.01, 1.24)	<0.001
Model 3	1.13 (1.04, 1.22)	0.005	1.20 (1.09, 1.32)	<0.001	1.16 (1,09, 1.23)	<0.001
Model 4	1.12 (1.04, 1.22)	0.005	1.20 (1.09, 1.31)	<0.001	1.16 (1.09, 1.23)	<0.001
Model 5	1.11 (1.02, 1.20)	0.02	1.16 (1.05, 1.29)	0.004	1.13 (1.06, 1.21)	<0.001
**Myocardial infarction**						
Unadjusted	1.15 (1.05, 1.26)	0.003	1.38 (1.20, 1.59)	<0.001	1.21 (1.12, 1.31)	<0.001
Age-adjusted	1.22 (1.11, 1.34)	<0.001	1.21 (1.06, 1.40)	0.006	1.23 (1.14, 1.33)	<0.001
Multivariable adjustment						
Model 1	1.16 (1.05, 1.28)	0.004	1.16 (1.01, 1.35)	0.049	1.16 (1.08, 1.27)	<0.001
Model 2	1.07 (0.95, 1.19)	0.25	1.09 (0.93, 1.27)	0.26	1.09 (0.99, 1.18)	0.09
Model 3	1.05 (0.94, 1.17)	0.3	1.10 (0.94, 1.29)	0.22	1.09 (0.10, 1.18)	0.06
Model 4	1.05 (0.95, 1.17)	0.3	1.09 (0.93, 1.27)	0.3	1.08 (0.99, 1.18)	0.09
Model 5	1.05 (0.94, 1.17)	0.4	1.06 (0.90, 1.25)	0.5	1.06 (0.97, 1.16)	0.19
**Ischemic stroke**						
Unadjusted	1.13 (1.01, 1.28)	0.049	1.37 (1.17, 1.61)	<0.001	1.21 (1.10, 1.33)	<0.001
Age-adjusted	1.23 (1.08, 1.40)	0.001	1.20 (1.03, 1.40)	0.02	1.22 (1.11, 1.35)	<0.001
Multivariable adjustment						
Model 1	1.25 (1.10, 1.42)	0.001	1.13 (0.95, 1.33)	0.17	1.19 (1.07, 1.31)	0.001
Model 2	1.29 (1.13, 1.47)	<0.001	1.13 (0.95, 1.34)	0.17	1.22 (1.09, 1.35)	<0.001
Model 3	1.28 (1.11, 1.46)	<0.001	1.13 (0.95, 1.35)	0.18	1.21 (1.09, 1.35)	<0.001
Model 4	1.28 (1.12, 1.47)	<0.001	1.12 (0.94, 1.34)	0.20	1.22 (1.09, 1.35)	<0.001
Model 5	1.31 (1.14, 1.50)	<0.001	1.13 (0.94, 1.36)	0.21	1.24 (1.11, 1.38)	<0.001

The Tromsø Study.

Model 1: Age, body mass index (BMI), systolic blood pressure (SBP) and diastolic blood pressure (DBP).

Model 2: Model 1 + HDL-cholesterol and total cholesterol.

Model 3: Model 2 + use of diuretics and other antihypertensive medication.

Model 4: Model 3 + current smoking and physical activity (physical activity ≥1 hr per week; yes = 1 / no = 0), current smoking (yes = 1 / no = 0).

Model 5: Model 4 + renal factors added (GFR the CKD-EPI and log ACR: GFR: CKD-EPI formula [17]. Log ACR: log albumin/creatinine ratio, mean of 3 urine samples). In analyses of men and women together, adjustment for gender is included in all models.

Number of events during follow up: 659 fatal- or non-fatal myocardial infarctions, 430 fatal- or non-fatal ischemic strokes, and 1433 deaths from all causes.

## Discussion

In this 12-15 year prospective study of 5700 men and women from the general population where persons with known diabetes or cardiovascular disease were excluded, a 1 SD (87 μmol/L) increase in serum uric acid was significantly associated with 31% increased risk for ischemic stroke in men, and all-cause mortality risk was increased in both genders, with 11% in men, and 16% in women, after multivariable adjustments. Gender-adjusted, multivariable analyses of pooled data from both men and women showed, for each 1 SD increase in serum uric acid, a 22% increased risk for ischemic stroke, and 13% increased risk for all-cause mortality.

The association between serum uric acid and ischemic stroke is in accordance with previous studies [5,19,20]. It has been suggested that serum uric acid may have harmful effects on platelet function [21] and cause endothelial dysfunction [22]. Vannorsdall et al. [23] reported that even a mild elevation of serum uric acid was associated with cerebral ischemia among community-dwelling adults. It was suggested that impaired vascular tone and endothelial dysfunction could contribute to ischemic changes, because they permit cerebrospinal fluid to cross the blood-brain barrier and cause areas of edema [23].

Quite opposite to this, it has been claimed that treatment with uric acid in combination with thrombolysis would be of benefit to patients suffering from acute stroke [24]. Uric acid is one of the most important endogenous antioxidants in the human brain, and high circulating uric acid could play a role against the deleterious effect of free radicals produced upstream in the synthesis of uric acid [24]. Accordingly, a J-curve relationship has been observed between serum uric acid and occurrence of ischemic stroke [19]. Our study did, however, not reveal any non-linear associations between serum uric acid and any of the endpoints. In women, serum uric acid lost significance as predictor for ischemic stroke when blood pressure and BMI were included as covariates. However, no gender interaction was observed. Differences in risk estimates for stroke between genders, may relate to gender-specific differences in vascular biology such as adaption to pro-inflammatory stress. Vlachopoulos et al. [25] reported that in newly diagnosed hypertensive persons, uric acid was associated with increased aortic stiffness in both genders, however a negative association with arterial wave reflection was observed only in women. Such differences like this in vascular function could influence the tendency of developing stroke. The Framingham study [11] also reported lack of independent association of uric acid with stroke, but this is in contrast to the gender specific analyses of the AMORIS-study [5]. However, BMI and use of antihypertensive medication was not accounted for in the AMORIS study [5].

We observed a significant association of serum uric acid with all-cause mortality, with a modest increase in mortality risk in both genders. In the Framingham study [11], no association was observed with all-cause mortality among the genders after adjustments for age, blood pressure, smoking, BMI, total cholesterol, intake of alcohol and medication. On the other hand, the NHANES study [3] reported a 13% increased mortality risk in women in fully adjusted analyses, but only non-significant associations in men. The fact that serum uric acid levels in women tend to increase during the fifth to the seventh decade due to postmenopausal reduction in uric acid excretion [2,26], whereas a flat (Figure 2) or slightly declining curve with ageing is seen in men [11], may influence the association with endpoints among the genders.

No independent association between increment in uric acid and MI was observed in the present study. However, significance did not depend on renal function or ACR as expected, but on lipids. Total- and HDL-cholesterol abolished the effect of uric acid on future MI. This is in consistence with a recent study [27] on the predictive value of uric acid on cardiovascular mortality among persons with type 2 diabetes in the general population. The inclusion of HDL-cholesterol, LDL-cholesterol, triglycerides, BMI, HbA1c and blood pressure as covariates abolished the significance of a 40% risk increment found in subjects with uric acid levels > 375 μmol/L in sex and age-adjusted analyses [27]. Subgroups with known diabetes or cardiovascular disease were excluded in the present study, as we aimed to examine the role of uric acid in a low-risk population. Subanalyses where persons with diabetes or preexisting cardiovascular disease were included, did not change our results. Neither did stratification according to uric acid values above or less than the upper reference range. This is in contrast to the NHANES study [3], where an increased risk of ischemic heart disease mortality was observed. Hazard ratios for each 59 μmol/l increase in uric acid level were reported to be 1.17 (95% CI 1.06-1.28) in men and 1.30 (95% CI 1.17-1.45) in women [3]. However, in that study, BMI was higher than in the present study, and covariates such as HDL-cholesterol and drug intake were not accounted for.

The Framingham Study [11] is one of the largest studies on the association of uric acid with CVD in the general population. Our study differs from the Framingham Study [11] in many ways. Mean age was 47 years in the Framingham study compared with 60 years in the present study, and thus mortality rate was lower in the Framingham study (12.4 per 1000 person years compared to 18.9 per 1000 person years). In general, studies that have failed to discern associations between uric acid and cardiovascular disease have had shorter observation time, included younger persons, and had low number of events per-person-years. As such they may have lacked the power to identify the contribution of hyperuricemia to cardiovascular outcomes [28]. The observation time in our study was longer than in most previous studies, and this may explain why we were able to detect associations in a study population where high-risk subjects had been excluded (e.g., subjects with diabetes and known CVD). It has been claimed that uric acid is a more important risk factor among Afro Americans than among Caucasians [3,11]. Our study shows that uric acid is a risk factor in the Caucasian population as well.

Several mechanisms could cause the uric acid metabolic pathway to be a CV risk factor. Uric acid may stimulate vascular smooth cell proliferation, and reduce vascular nitric oxide production. For a review, see [29]. The action of xanthine oxidase leads to generation of superoxide anions [30]. This could mean that xantine oxidase activity is the key risk factor, with uric acid just an epiphenomenon. Uric acid per se has been described as a scavenger with antioxidant effects [29-32]. It is noteworthy that some preliminary intervention studies have shown that the xanthine oxidase inhibitor Allopurinol lowered blood pressure in hypertensive adolescents [33], and had anti-ischemic effects in patients with angina pectoris [34]. Allopurinol also reduced cardiovascular and hospitalization risk in a small study of patients with renal failure [35]. A recent study found that hyperuricemia was significantly associated with poor outcomes in heart failure patients without chronic kidney disease, but not in hyperuricemic persons with renal failure [36]. The latter could suggest that hyperuricemia may predict poor outcomes primarily as a marker of xanthine oxidase activity, and not due to impaired renal excretion of uric acid. Future intervention studies will hopefully explore this important issue further.

A major shortcoming of our study is that serum uric acid was done as a single measurement. Furthermore, assessment was done in a population of Caucasians only, so our results may not pertain to other ethnical groups. The study is strengthened by the solid attendance rate, a long follow-up time, the thorough validation of endpoints, and the ability to correct for confounding risk factors such as renal function, ACR, traditional cardiovascular risk factors and the use of antihypertensive medication and diuretics.

## Conclusion

After multivariable adjustment, serum uric acid was significantly associated with increased risk of future ischemic stroke in men and with all-cause mortality in both genders. Associations of uric acid with myocardial infarction lost significance after adjustments for lipids. We conclude that serum uric acid is an independent marker of ischemic stroke in men, and all-cause mortality in both genders in a Caucasian, general population. Gender-specific analyses should be given priority in future studies.

## Competing interests

The authors have no conflict of interest to disclose related to the present study.

## Authors’ contributions

Study design: HMS, IT, TJ. Data collection: MLL. Data analyses: HMS, IT, TJ, BOE. Writing the first draft: HMS, IT, TJ. Data interpretation, discussion and preparation of the final manuscript: HMS, IT, JVN, BOE, MLL, MDS, SNZ, SW, SC, TJ. All authors read and approved the final manuscript.

## Pre-publication history

The pre-publication history for this paper can be accessed here:

http://www.biomedcentral.com/1471-2261/13/115/prepub
